# P-637. Underlying Medical Conditions in Children 8 to <20 Months of Age Hospitalized with Respiratory Syncytial Virus

**DOI:** 10.1093/ofid/ofaf695.850

**Published:** 2026-01-11

**Authors:** Julia Bratic, Tess Stopczynski, Leila C Sahni, Pedro A Piedra, Laura S Stewart, Marian G Michaels, John Williams, Rangaraj Selvarangan, Jennifer E Schuster, Daniel C Payne, Mary A Staat, Eileen J Klein, Janet A Englund, Geoffrey A Weinberg, Peter G Szilagyi, Ariana Toepfer, Heidi L Moline, Natasha B Halasa, Julie A Boom

**Affiliations:** Texas Children's Hospital, Baylor College of Medicine, Austin, TX; Vanderbilt University Medical Center, Nashville, Tennessee; Baylor College of Medicine and Texas Children's Hospital, Houston, Texas; Baylor College of Medicine, Houston, Texas; Vanderbilt University School of Medicine, Nashville, Tennessee; University of Pittsburgh/ CHP, Pittsburgh, Pennsylvania; University of Wisconsin, Madison, Wisconsin; Children’s Mercy Hospital, Kansas City, Missouri; Children's Mercy Kansas City, Kansas City, MO; Cincinnati Children's Hospital Medical Center, Decatur, GA 30030-3637, Georgia; Cincinnati Children's Hospital Medical Center, Decatur, GA 30030-3637, Georgia; Seattle Children's Hospital and University of Washington School of Medicine, Seatte, Washington; Seattle Children’s Hospital/Univ. Washington, Seattle, Washington; University of Rochester Sch Med & Dent, Rochester, New York; UCLA, Los Angeles, California; 15CDC, Atlanta, Georgia; US-CDC, Atlanta, Georgia; Vanderbilt University Medical Center, Nashville, Tennessee; Baylor College of Medicine, Houston, Texas

## Abstract

**Background:**

Nirsevimab, a highly effective monoclonal antibody, is recommended for all infants < 8 months of age and high-risk patients 8 to < 20 months of age with chronic lung disease (CLD) of prematurity, cystic fibrosis (CF), severe immunocompromise or who are American Indian/Alaska Native (AI/AN) to prevent RSV.^1,2^ Other children with underlying medical conditions (UMCs) may also benefit from nirsevimab. We characterized the clinical course of RSV-associated hospitalization in children with UMCs by nirsevimab eligibility.

**Figure 1.**
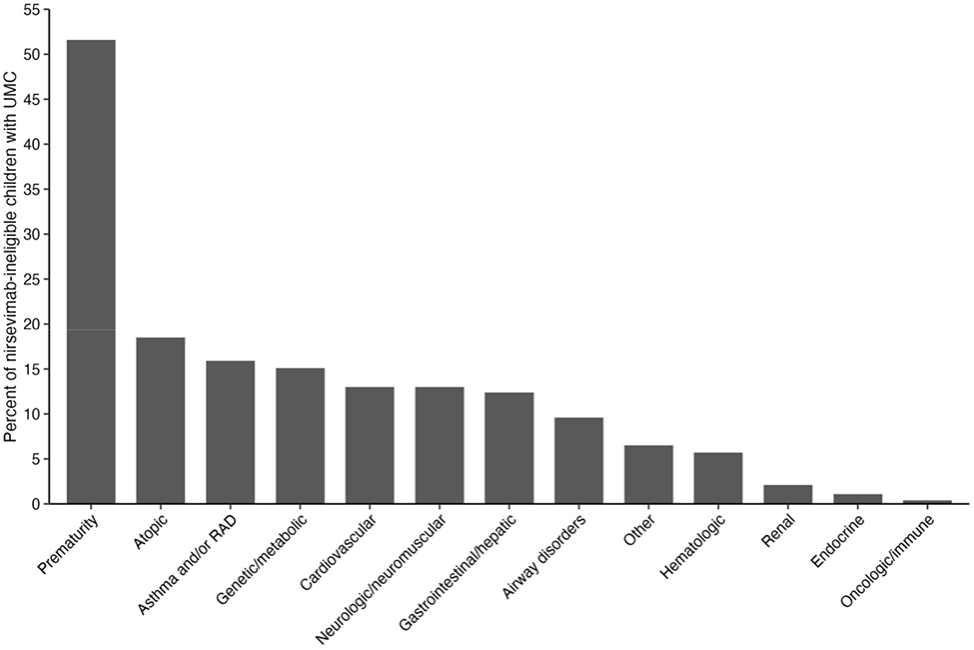
Underlying medical conditions1 for hospitalized children with at least one underlying medical condition aged 8 to <20 months ineligible for nirsevimab (n=523, 12/01/2016 to 07/16/2023). 1Prematurity defined as <37 weeks gestation. Atopic conditions include atopic/allergic conditions not including asthma/RAD. Genetic/metabolic conditions include development disorders (autism spectrum disorder, global developmental delay, pervasive development disorder, intellectual disability, gross motor delay, other developmental disorder) and genetic conditions (down syndrome, congenital syndromes, inborn errors of metabolism, disorders of fatty acid metabolism, chromosomal deletion/duplication/translocation, other genetic syndrome). Cardiovascular conditions include congenital heart malformations and heart conditions. Neurologic/neuromuscular conditions include cerebral palsy, seizure disorder, and other neurologic/neuromuscular conditions (e.g., autonomic dysfunction, agenesis, blindness, muscular dystrophy, severe scoliosis). Gastrointestinal/hepatic conditions include chronic liver disease, surgical/congenital GI disease, inflammatory bowel disease, eosinophilic esophagitis, pancreatic insufficiency, and other gastrointestinal conditions. Airway disorders include, e.g., tracheostomy dependence, tracheomalacia, cleft lip/palate, vocal cord paralysis, etc. Other conditions include other significant medical conditions requiring chronic treatment. Hematologic conditions include sickle cell disease, other anemias, coagulopathy, arterial or venous thromboses, thrombocytopenia, and other blood disorders. Renal conditions include chronic renal failure, end stage renal disease, chronic nephritis, nephrotic syndrome, and other kidney conditions. Endocrine conditions include diabetes mellitus, adrenal insufficiency, hypothyroidism or hyperthyroidism, and other endocrine conditions. Oncologic/immune conditions include cancer and undergoing treatment, transplant recipient, asplenia, rheumatologic disease, primary immunodeficiency, and other immune conditions.

**Table 1.**
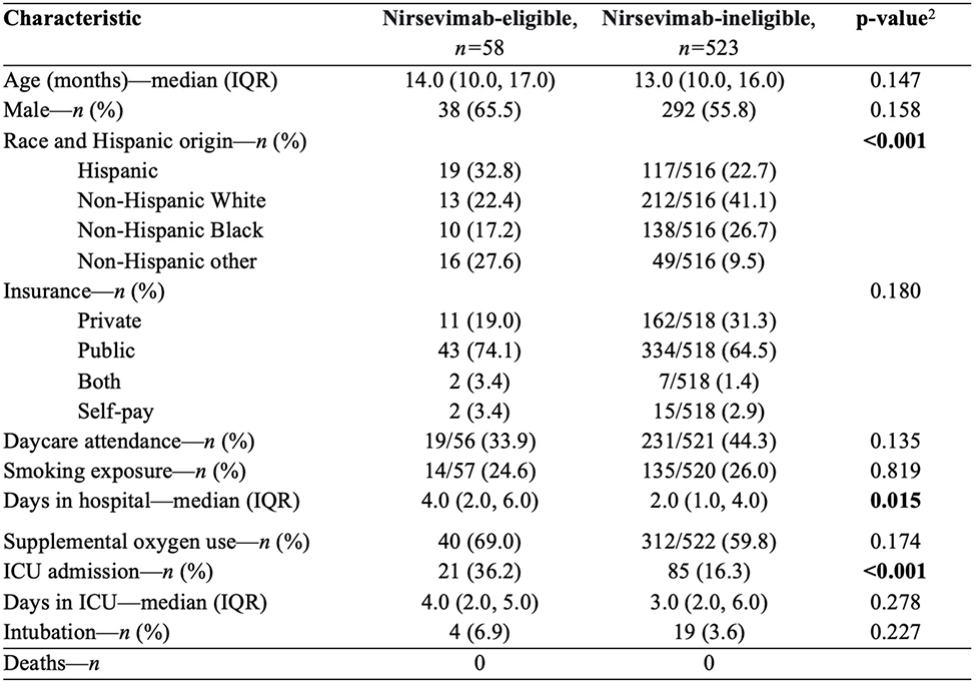
Children with an underlying medical condition 8 to <20 months hospitalized with RSV (N=581), stratified by eligibility for nirsevimab1 (12/01/2016 to 07/16/2023). Abbreviations: IQR, interquartile range; ICU, intensive care unit. 1Eligibility defined as children 8 to<20 months old with CF, CLD of prematurity, or immunocompromising condition, or AI/AN children. 2p-values calculated by Pearson’s χ² test for categorical variables and the two-sample t-test with unequal variances for continuous variables.

**Methods:**

Data were obtained by the New Vaccine Surveillance Network (NVSN; a 7-site, prospective surveillance platform of acute respiratory illness) from December 1, 2016 through July 16, 2023. Analysis was restricted to hospitalized children 8 to < 20 months with a positive RT-PCR for RSV, and p-values were calculated by Pearson’s χ² test for categorical variables and the two-sample t-test with unequal variances for continuous variables. Clinical history and UMCs were obtained by parent/guardian interview and/or chart review. We stratified by eligibility for nirsevimab, which was broadly defined as AI/AN, those with CLD of prematurity, CF, and/or immunocompromise.

**Results:**

A total of 581 children 8 to < 20 months with UMCs were hospitalized with RSV. Of these, 58 (10%) were nirsevimab-eligible (Table 1), including 55.2% with CLD of prematurity, 12.1% with immunocompromise, and 6.9% with CF; 22.4% were AI/AN and 3.4% were AI/AN with CLD of prematurity. Compared with nirsevimab-ineligible children, nirsevimab-eligible children had longer duration of hospitalization and higher frequency of ICU admission (Table 1, p=0.015 and p< 0.001, respectively); notably, 80% of ICU admissions were nirsevimab-ineligible children. Of 523 (90%) nirsevimab-ineligible children, the majority (51.6%) were premature (median gestational age 34 weeks (IQR: 32, 36) (Figure 1) vs. 26 weeks (IQR: 25, 28.8) in nirsevimab-eligible children). Other UMCs were not as commonly present as prematurity.

**Conclusion:**

Most hospitalized RSV-positive children 8 to < 20 months of age with UMCs were not eligible for nirsevimab and most ineligible children were premature. Additional analyses are needed to further characterize children in their second RSV season who are at highest risk for severe RSV disease.

**Disclosures:**

Pedro A. Piedra, MD, Gilead: Honoraria|GSK: Grant/Research Support|Icosavax: Grant/Research Support|Merck: Advisor/Consultant|Merck: Grant/Research Support|Moderna: Advisor/Consultant|Novavax: Grant/Research Support|Pfizer: Advisor/Consultant|Sanofi-Pasteur: Advisor/Consultant|Sanofi-Pasteur: Grant/Research Support|Shionogi: Advisor/Consultant|Shionogi: Grant/Research Support Marian G. Michaels, MD, MPH, Merck: Grant/Research Support Rangaraj Selvarangan, PhD, Altona: Grant/Research Support|Biomerieux: Advisor/Consultant|Biomerieux: Grant/Research Support|Biomerieux: Honoraria|Cepheid: Grant/Research Support|Hologic: Grant/Research Support|Hologic: Honoraria|Meridian: Grant/Research Support|Qiagen: Grant/Research Support Daniel C. Payne, PhD, MSPH, Merck: Advisor/Consultant|Moderna: Advisor/Consultant Mary A. Staat, MD, MPH, Centers for Disease Control and Prevention: Grant/Research Support|Cepheid: Grant/Research Support|Merck: Advisor/Consultant|Merck: Grant/Research Support|National Institutes of Health: Grant/Research Support|Up-To-Date: Royalties Janet A. Englund, MD, AstraZeneca: Board Member|AstraZeneca: Grant/Research Support|Cidarra: Member Data Safety Monitoring Board|GlaxoSmithKline: Advisor/Consultant|GlaxoSmithKline: Grant/Research Support|Meissa Vaccines: Advisor/Consultant|Merck: Advisor/Consultant|Merck: Grant/Research Support|Moderna: Advisor/Consultant|Moderna: Grant/Research Support|Pfizer: Advisor/Consultant|Pfizer: Grant/Research Support|Shionogi: Grant/Research Support Geoffrey A. Weinberg, MD, Inhalon Biopharma: Advisor/Consultant|Merck & Co: Honoraria Natasha B. Halasa, MD, CSL-Seqirus: Advisor/Consultant|Merck: Grant/Research Support

